# An Enjoyable Workplace Combined Exercise Program for Health Promotion in Trained Employees: Yoga, Pilates, and Circuit Strength Training

**DOI:** 10.3390/sports11040084

**Published:** 2023-04-14

**Authors:** Konstantina Karatrantou, Christos Batatolis, Petros Chatzigiannis, Theodora Vasilopoulou, Anastasia Melissopoulou, Panagiotis Ioakimidis, Vassilis Gerodimos

**Affiliations:** Department of Physical Education and Sport Science, University of Thessaly, 42100 Trikala, Greece

**Keywords:** office workers, wellness, musculoskeletal pains, functional capacity, physical fitness

## Abstract

Corporate wellness has become an important public health priority through the designing and implementation of different workplace exercise interventions. The objectives of this study were to investigate: (a) the effectiveness of a 4-month workplace combined yoga, Pilates, and circuit strength training program (outside work shift) on health indices, functional capacity, and physical fitness in office employees; and (b) the employees’ enjoyment following the program. Fifty physically active office employees (26–55 years old) were equally divided into training (TG) and control groups (CG). The TG followed a 4-month (3 times/week, 50–60 min/training) combined yoga, Pilates, and circuit strength training program. Health indices (body composition, body mass, circumferences, musculoskeletal pains), functional capacity (flexibility, balance), and physical fitness (strength, aerobic capacity) were measured before and after the 4-month time period. After the completion of the program, the TG participants’ enjoyment was assessed. The TG significantly improved (*p* < 0.001) all health, functional capacity (11.3–82.0%), and physical fitness indices (33.9%), except for aerobic capacity, which did not change (*p* > 0.05). Furthermore, a great percentage of employees (84%) reported high levels of enjoyment. This program could be effectively and safely used in workplace settings as an enjoyable intervention to improve specific health, functional capacity, and physical fitness indices in office employees.

## 1. Introduction

The modern lifestyle and technological advances have improved people’s lives and livelihoods, but with unexpected consequences to their health and well-being [1]. In the age of technology, an average day for many people involves extended periods of sedentary behaviors that require very low energy expenditure (of less than 1.5 metabolic equivalents) and typically involve lying down and sitting [2]. Sedentary and other unhealthy behaviors have been characterized as some of the biggest health hazards of this generation, and they are especially common in the workplace setting (i.e., office employees) [3,4]. Indeed, a great percentage of employees spend a lot of time sitting in the office (>70% of the work time), adopt unhealthy behaviors (e.g., physical inactivity, smoking, alcohol, excessive caffeine consumption, unhealthy eating habits), and suffer from serious physical and mental health-related problems [5,6,7]. The adoption of a “sedentary and unhealthy lifestyle” in conjunction with demanding and often exhausting working hours and unsuitable working conditions that prevail in different work settings is associated with depressed wages and earnings, reduced workforce participation and labor productivity, and increased rates of early retirement and disability [4,8]. The design and implementation of workplace exercise programs, beyond the benefits for the employees themselves, have future financial benefits for the companies and employers. Indeed, previous studies that implemented workplace wellness programs in different companies reported: (a) medical savings of up to 59%; (b) improvement of employee morale by 77%; (c) creation of a healthy workforce (75%); (d) reduction in early retirement and absences from work due to illness, injury, and disability; and (e) productivity gains between 41–65% [9,10]. Additionally, a previous study reported that for every dollar spent on workplace wellness programs, the future investment for the company itself is approximately USD 6, reducing medical costs by approximately USD 3.27 and costs associated with absenteeism from work by approximately USD 2.73 [11].

For this reason, the creation and nurturing of a “corporate wellness philosophy” have become important from a public health perspective, achieved through the designing, implementation, and guidance of different workplace wellness interventions during or outside the work shift [4,12,13]. In more detail, many companies tried to promote employees’ physical activity by using pedometers recording daily steps at work and ergo-desks whose bases were bikes or treadmills [14,15]. Beyond strategies to increase physical activity and wellness in work settings, exercise programs (e.g., team sports, weight training, stretching, yoga, Pilates) during or outside (before or after the working hours) of the work shift are used to improve different features of physical and mental health, musculoskeletal pain, functional capacity, and physical fitness [13,16,17,18,19,20,21,22,23,24,25,26,27,28]. Although a great proportion of the aforementioned studies in the workplace implemented various strength training programs for different muscle groups, using exercises with body weight, small portable equipment, or strength machines [13,17,20,23,25,26], there is little research emphasizing circuit strength training [29,30,31]. However, during the last decade, circuit strength training has grown in popularity as a time-efficient (decreasing total training time by around two-thirds), effective, and pleasant form of strength training [32], and could be an alternative exercise approach for employees who have increased working obligations and limited free time. Furthermore, yoga and Pilates are two of the most widespread mind–body exercise modalities (combining body movement, mental focus, and controlled breathing), widely used in different workplace wellness interventions for physical (decreasing musculoskeletal pains, increasing functional capacity indices, etc.) and mental health promotion (decreasing anxiety, increasing sense of self-control and wellbeing, etc.) [27,28,33].

One of the most common problems that we face when implementing exercise programs is dropping out of the program due to the lack of enjoyment and the monotony of training [34,35]. There is evidence that the promotion of positive feelings during exercise is one of the most important strategies to increase systematic participation in exercise interventions [34,35]. The creation of positive feelings and high levels of enjoyment and satisfaction during the program are associated with increased levels of exercise adherence [34,35]. A multimodal exercise program that combines different training goals or different modes of exercise every week may be an efficient, enjoyable, and interesting intervention for health promotion in the workplace. Although several studies implemented different combined exercise programs for employees during or outside the work shift, few of them evaluated the participants’ enjoyment following the program [13,26], which is very important for its sustainability.

Taking all the above into consideration, we designed a supervised combined multimodal exercise program for office employees, which was performed at the gym of the workplace building before or after the work shift, according to the employee’s obligations and responsibilities. The implementation of workplace exercise programs outside the work shift (compared to workplace programs during the work shift) secures the successful participation of the employees, without interfering with the proper functioning of the company. Thus, employees with increased responsibilities and without the appropriate breaks during the work shift probably found that it was easier to follow an offered workplace program outside the working hours. Therefore, the main objectives of the present study were to investigate: (a) the efficiency of a 4-month workplace (outside the work shift) combined yoga, Pilates, and circuit strength training program on different health indices, musculoskeletal pains, functional capacity (flexibility, balance), and physical fitness indices (strength, aerobic capacity); and (b) the employees’ enjoyment following the combined exercise program. We chose to incorporate into the program three of the most popular modes of exercise all over the world, “yoga, Pilates, and circuit strength training”, which can be easily modified and integrated into one combined training program in the workplace. It is expected that the combined yoga, Pilates, and circuit strength training program that will be implemented could be an enjoyable, safe, and effective best practice to improve specific health, functional capacity, and physical fitness indices of office employees.

## 2. Materials and Methods

### 2.1. Participants

Fifty office employees (seventeen males, thirty-three females) volunteered to participate in the present study. After the baseline measurements, the participants were divided into two equal groups: the training group (TG; *n* = 25) and the control group (CG; *n* = 25) (Table 1). All the participants were administrative office staff who spend at least 7 h/day (5 days per week; ≥35 h per week) working in the office. The participants were healthy and free of any illness, disease, or injury and did not report the use of any medication. The participants of both the TG and CG were physically active and participated 2–3 times per week (1 h/day) in non-organized physical activities, such as the use of bicycle for daily transportation, the use of stairs instead of the elevator, and the active participation in household and outside home chores. Both the TG and CG maintained these non-organized physical activities throughout the 4-month period, as this was evaluated before and at the end of the 4-month period using a specific physical activity questionnaire. Furthermore, the participants in the TG had previous experience in systematic exercise at least 1 year before the start of the study. Before the initiation of the study, the participants were (a) informed about the experimental procedures, (b) signed an informed consent form, (c) completed a standardized health history questionnaire by the American College of Sports Medicine (ACSM) to assess their health and/or activity status, and (d) adduced a health certificate given by their doctor. The Ethics Committee of the University of Thessaly approved the study.

### 2.2. Study Design

Initially, a pilot study was carried out to determine the final testing and training procedures. One week before the start of the study, the participants were familiarized with the experimental testing and training procedures. Subsequently, the baseline measurements were performed in the total sample. Completing the pretraining testing, the participants were assigned to either a training group (TG, *n* = 25) or a control group (CG, *n* = 25). The TG participated 3 times per week in the combined training program, while the CG did not perform any systematically organized training program during the 4 months. It should be mentioned that no adverse effects or injuries were reported during the study both in the TG and in the CG. After the end of the 4 months, both the TG and the CG repeated the pretraining measurements in the same order and at the same time of the day. All measurements were performed by the same investigator who was blinded regarding the type of training protocol and the allocation of the participants in the two groups. The training program was supervised by an exercise instructor with special expertise in circuit, yoga, and Pilates training programs for health promotion. During the 4-month training period, special exercise attendance books were completed to ensure participants’ exercise adherence (all the participants of the study performed 48 training sessions in total). The exercise adherence was also strengthened using three motivation strategies: (a) think positive, (b) make a commitment, and (c) set short-term goals [36].

### 2.3. Training Program

The description of the training program of this study followed the instructions of the Consensus on Exercise Reporting Template (CERT) developed by Slade et al. [37] (Supplementary Table S1). The CERT is a 16-item checklist designed and developed by an international panel of exercise experts to improve the reporting of exercise programs in all evaluative study designs and contains 7 categories: materials, provider, delivery, location, dosage, tailoring, and compliance [37].

In the present study, the TG participated 3 days a week, in a 4-month (48 training sessions in total) combined yoga, Pilates, and circuit strength training program. The training program took place in small groups (4–5 participants per group) at the organized gym of the workplace building. The training sessions were performed outside the work shift (immediately before or after the work shift) and an exercise instructor supervised them. The participants performed 2 training sessions per week of strength training using a circuit training program and 1 training session per week of flexibility and balance using yoga–Pilates training. Every training session lasted between 50–60 min (15-min warm-up, 30–40-min main part, and 5-min cool down).

In more detail, the main part of the training program included:

(a) Flexibility and balance training: The flexibility and balance training (frequency: 1 time/wk) included yoga and Pilates exercises for the whole body. The program included routines—sequences using exercises from different positions such as upright, supine, prone, side-lying, using body weight, and swiss balls (Supplementary Table S2). The duration of every exercise/pose was 15–30 s (for yoga poses) or 12–20 repetitions/15–30 s (for Pilates exercises) and the number of sets ranged from 2 to 4.
(b) Strength training: The strength training program consisted of a circuit training program with 3–4 rounds. Every round lasted approximately 10–12 min and included 7–8 polyarticular exercises per day using body weight, TRX, medicine ball, and dumbbells (Supplementary Table S2). The duration of every exercise was 20–30 s. After the end of every round, the participants rested for 1–2 min.

The training load characteristics during the 4-month intervention were progressively increased according to the recommendation of the ACSM [38]. The gradual increase (per month) in the training load during flexibility, balance, and strength exercise workouts throughout the 4-month period is analytically presented in Table 2.

Additionally, an indicative training session (the 10th training session) of the 4-month intervention program is analytically presented in Table 3.

Before the start of the study, all the participants had similar levels (they were of an advanced level, according to their baseline measurements, which were compared with scientific norms) of functional capacity and physical fitness. The exercise programs that were selected and used in the present study were similar for all the participants and were based on their initial functional capacity and physical fitness levels. However, there was some degree of tailoring in the training load characteristics according to the level of each individual. In more detail, in the exercises where there was external resistance (i.e., dumbbells, medicine ball), this was adjusted according to the individual level of the participants so that they could get the required time or repetitions per exercise. In the exercises where there was no external resistance, there was a small adjustment of the duration (approximately 4–5 s) or repetitions (approximately 3–4 repetitions) per exercise according to the individual level of the participants.

### 2.4. Testing Procedures

Health indices, musculoskeletal pains, functional capacity, and physical fitness were measured, in both the TG and CG, before and after the 4 months.

#### 2.4.1. Health Indices

✓ Body mass and body height were measured using a calibrated physician’s scale (Seca model 755, Seca, Hamburg, Germany) and a telescopic height rod (Seca model 220, Seca, Hamburg, Germany), respectively, according to the recommendations of the ACSM [39].
✓ Waist and hip circumferences were measured using a conventional measuring tape (Seca 201, Seca, Hamburg, Germany), as previously described by the ACSM [39].
✓ Body composition (percentage of body fat—% BF) was assessed using the bioelectrical impedance method (Maltron 900) [39].
✓ Musculoskeletal pains and discomforts (duration and intensity of pain, days off, and difficulties in daily activities due to musculoskeletal pains) were evaluated in 9 body parts (neck, shoulders, elbows, wrists/hands, upper back, lower back, hip, knees, foot/ankle) using the Nordic questionnaire [40].

#### 2.4.2. Functional Capacity Indices

✓ Flexibility: Lower back and hamstring flexibility was measured with the sit and reach test using a Flex-Tester box (Novel Products Inc., Rockton, IL, USA), and shoulder range of motion was measured with a back scratch test using a measuring tape according to the recommendations of the ACSM [39]. The participants performed 3 maximal trials during each test, and the best score was considered for analysis.
✓ Balance: The static balance was assessed for both legs using the single-limb stance test with eyes open, as previously described by Rinne et al. [41]. The participants performed 3 trials for each leg, and the average time (in s) of the 3 trials was considered for analysis. Dynamic balance was evaluated using the timed up-and-go test (TUG), as previously described by Rikli and Jones [42]. Following 2 familiarization trials, the participants performed 3 maximal trials with rest periods of 30 s, and the best time (in s) was used to evaluate performance.

#### 2.4.3. Physical Fitness Indices

✓ Strength: Muscular endurance of the chest and triceps muscles was assessed using the push-up test according to the ACSM’s instructions [39]. The participants were instructed to do as many repetitions as they could, and the maximal number was then used for analysis.
✓ Aerobic capacity: The YMCA 3-min step test was used to assess aerobic capacity. The participant stepped up and down on a 30-cm box for a total time of 3 min following the metronome cadence, which was set at 96 beats per minute (4 steps per cycle). The participants’ HR was measured 1 min following the termination of the step test and considered for analysis [13].

#### 2.4.4. Enjoyment

The participants’ enjoyment following the workplace combined yoga, Pilates, and circuit strength training program was assessed using the subscale of the intrinsic motivation questionnaire created by Mc Auleys et al. [43]. The subscale for enjoyment assessment includes 4 questions, which are scored using a 5-point Likert scale with 1-step increments (from 1, absolutely disagree, to 5, absolutely agree). The questions for the assessment of the participants’ enjoyment were the following: (1) “I enjoyed the activities of the combined workplace training program.” (2) “I thought that the combined workplace training program was interesting.” (3) “I thought that the time passed very quickly when I participated in the combined workplace training program.” (4) “When I participated in the combined workplace training program, I enjoyed it.” The score of each question as well as the mean overall score (from all questions) were used to analyze the data.

### 2.5. Statistical Analysis

In our study, all statistical analyses were performed using IBM SPSS Statistics v.26 software (IBM Corporation, Armonk, NY, USA), and the results are presented as means ± standard deviations. A statistical power analysis (software package GPower 3.0) before the initiation of the study indicated that a total number of 40 participants (20 participants in each group) would yield adequate power (>0.85) and a level of significance (<0.05). The total sample of the present study was fifty office employees (twenty-five participants in each group). The normality of the data was examined, separately for each group, using the Shapiro–Wilk test (all variables followed the normal distribution). Two-way analysis of variance (ANOVA), 2 groups (TG and CG) × 2-time points (pre and post-training), with repeated measures on the “time point” factor and multiple comparisons with the Sidak method were used to analyze the data. The Cohen’s effect sizes were calculated using the equation: d = difference between means/pooled SDs. One-way ANOVAs were used between groups to compare the relative changes from pre- to post-training in all tested parameters. The significance level was set at *p* < 0.05.

## 3. Results

### 3.1. Health Indices

Analyses of variances showed significant “group × time” interaction effects on body mass, percentage of body fat, as well as on waist and hip body circumferences (*p* < 0.01). Specifically, in the TG all the above health indices were significantly lower at post-training versus pre-training measurements (*p* < 0.01; d = 0.16–0.37); while in the CG, all the above variables did not change (Table 4). Comparisons between groups revealed that all post-training values were significantly lower in the TG versus the CG (*p* < 0.01; d = 0.10–0.33), while the pre-training values did not differ between the two groups (*p* > 0.05).

### 3.2. Musculoskeletal Pain Indices

Analyses of variances showed significant “group × time” interaction effects on all the musculoskeletal pain indicators (*p* < 0.001). Especially, the duration and intensity of pain, the absenteeism from work, and the negative impact of musculoskeletal pains in daily activities were significantly lower at post-training versus the respective pre-training values in the TG (*p* < 0.001; d = 1.08 = 1.50). In the CG, all the above indicators did not change. Comparisons between groups revealed that all post-training values were significantly lower in the TG versus the CG (*p* < 0.001; d = 1–1.30), while the pre-training values did not differ between the two groups (*p* > 0.05; Table 5).

### 3.3. Functional Capacity Indices

Significant “group × time” interaction effects were observed for flexibility, static, and dynamic balance. In the TG, flexibility of lower (d = 1) and upper limbs (d = 0.54–0.65), static balance (d = 1.28–1.84), and dynamic balance (d = 0.55) values were significantly improved after the training program compared with the pre-training values (*p* < 0.001). In the CG, flexibility, static, and dynamic balance values did not change (Table 6). Comparisons between groups revealed that all post-training values were significantly improved in the TG versus the CG (*p* < 0.001; d = 0.35–1.78), while the pre-training values did not differ between the two groups (*p* > 0.05; Table 6).

### 3.4. Physical Fitness Indices

✓ Strength endurance: A significant “group × time” interaction effect was observed for strength endurance. In the TG, push-up test values were significantly improved after the training program (mean change: 33.9%) compared with the pre-training values (*p* < 0.001; d = 0.44; pre-training: 7.8 ± 6.6 reps; post-training: 11.0 ± 7.6 reps). In the CG, strength values did not change (pre-training: 7.9 ± 6.5 reps; post-training: 8.2 ± 6.6 reps). Comparisons between groups revealed that post-training values were significantly improved in the TG versus the CG (*p* < 0.001; d = 0.39), while the pre-training values did not differ between the two groups (*p* > 0.05).
✓ Cardiorespiratory fitness: A non-significant “group × time” interaction effect was observed for cardiorespiratory fitness (*p* > 0.05). Post-training values did not change after the training program in the TG (pre: 114.3 ± 13.1 beats/min and post: 114 ± 12.6 beats/min) and CG (pre: 114.7 ± 15.7 beats/min and post: 113.1 ± 15 beats/min).

### 3.5. Enjoyment

According to the results of the study, most of the participants (84%) stated high levels of enjoyment following the 4-month workplace combined training program (question 1: 4.4 ± 0.7 score, question 2: 4.7 ± 0.5 score, question 3: 4.1 ± 0.9 score, question 4: 4.4 ± 0.8 score, total score from all questions: 4.4 ± 0.6).

## 4. Discussion

Over the last few decades, corporate wellness programs have intended to promote and boost a wide-ranging approach to employee health by adopting a health-conscious corporate culture [4]. More and more companies all over the world offer different educational, practical, and/or evidence-based workplace wellness interventions, during or out of the work shift, demonstrating promising results in different features of physical and mental health [4]. This study designed and implemented with success a supervised multimodal exercise program, used out of the work shift (before or after the working hours), that combined two training sessions of circuit strength training and one training session of yoga–Pilates training per week. Τhe timetable of the program was flexible and organized according to the employees working obligations. The flexible timetable of the program assisted the participants to implement successfully the 4-month training program without dropping out and without affecting the smooth operation of the company. In contrast, another study, which examined the efficacy of a workplace exercise program for hospital workers, revealed low levels of exercise adherence (increased dropout levels) due to the incompatibility of the intervention with working obligations [44]. 

This study indicated that a 4-month (3 days/week) workplace combined yoga, Pilates, and circuit strength training program decreased body fat, body mass, body circumferences, and musculoskeletal pains and improved functional capacity (flexibility, static, and dynamic balance) and strength endurance in trained office employees. The results of this study are in accordance with previous studies that investigated the effects of specialized workplace exercise programs or other exercise programs outside the workplace reporting an improvement in various indices of functional capacity, physical fitness (strength), musculoskeletal pains, and health [13,16,17,18,19,20,22,23,26,45,46]. Nevertheless, the total amount of improvement revealed in this study in several indicators is lower than in some other studies [13,16,17,20,23,26,45,46]. The divergence in the total amount of improvement of our study and the previous studies may be correlated with different independent and/or interactive factors such as the training load (i.e., training frequency), the physical activities/exercises, and/or the subjects’ characteristics (physical fitness level). For instance, there is evidence that an exercise program provokes greater adaptations in untrained/beginners than in physically active or trained individuals. In previous studies, the participants were untrained with low levels of functional capacity and physical fitness or with musculoskeletal pains in different body parts and/or were older in chronological age [13,16,17,20,23,26,45,46,47], while in the present study, the participants were physically active, with at least 1 year of experience with organized exercise. Beyond the differences in subject characteristics, an additional factor that may account for the incompatible results (regarding the total amount of improvement) between our study and some of the previous studies is the training characteristics. The findings of previous studies indicated that greater training frequency per week causes greater adaptations to the human body. In the present study, the participants exercised 3 times/week, whereas in some other studies that implemented workplace interventions and reported greater results, the participants exercised above 3 times/week [13,26,47]. We also mentioned that the control group did not change any of the measured health, functional capacity, and physical fitness parameters. As we mentioned in the methods section, the participants of both the TG and the CG during the 4-month intervention participated 2–3 times per week (1 h/day) in non-organized physical activities (i.e., use of bicycle for daily transportation, use of stairs instead of elevator, active participation in household and outside home chores). It seems that, in the control group, the daily non-organized physical activities by themselves were not capable of causing significant improvements in the health, functional capacity, and physical fitness parameters. The results of this study regarding the control group strengthen the guidelines of the American College of Sports Medicine, which recommend the incorporation of organized exercise programs as the most effective approach for physical and mental health benefits [38,39].

An important finding of this study is that the combined exercise program did not affect cardiorespiratory fitness in our trained office employees. It should be mentioned that our combined exercise program did not include a specific aerobic exercise modality to induce immediate responses in cardiorespiratory fitness. However, some previous studies, which also did not consist of specific aerobic exercise modalities, reported positive effects on cardiorespiratory fitness following a circuit training program [29,31]. As previously mentioned in the lower magnitude of neuromuscular adaptations, the participants’ training levels may account for the conflicting results between this and some of the previous studies [29,31], which reported improvements in aerobic capacity following a circuit training program. The participants of this study had a good level of physical fitness, in contrast with other studies, whose participants were beginners; as a result, the circuit training provoked some improvements in aerobic capacity [29,31]. Therefore, it seems that people with a better background in training may need more specific training stimulus for improving cardiorespiratory fitness. Previous studies that implemented workplace exercise programs using different specific aerobic exercise modalities (i.e., brisk walking–running, aerobic dance, zumba) reported significant improvements in the aerobic capacity of employees [13,17,22,26,29,48].

Even though the systematic participation of adult population in organized physical activities is associated with various physical and mental health benefits, a significant percentage of them drop out of the program during the first six months [34,35] due to the increased obligations as well a lack of enjoyment and the monotony of the exercise program. A multimodal exercise program that combines different exercise modalities may be an alternative and interesting practice and perhaps the best method to reduce the monotony of training and increase positive feelings during exercise, increasing exercise adherence. The results of the present study showed that a great percentage of office employees were absolutely fulfilled with their participation in the combined circuit, yoga, and Pilates training program. In the literature, although several studies designed and implemented different combined and multimodal exercise programs for healthy adults inside or outside the workplace to improve various indices of functional capacity and physical fitness, few of them evaluated the participants’ enjoyment following the program [13,26,35,49]. The results of the above studies have also reported high levels of enjoyment following a workplace wellness program. From the results of the present study, it seems that a flexible schedule in the implementation of a workplace wellness program according to the employees working obligations and in conjunction with the high levels of enjoyment, may be an efficient strategy to increase exercise adherence, thus minimizing the percentage of dropouts.

The present study has some limitations that could affect its outcomes and as a result their generalization. Firstly, the findings of this study are limited to the design, implementation, and evaluation of a 4-month workplace combined exercise program, undertaken outside the work shift (before or after the working hours according to the employee’s obligations) and including strength training using a circuit exercise program as well as flexibility and balance training using yoga–Pilates workouts. Future studies should compare the efficiency and safety of equivalent exercise programs in other target groups with different characteristics (i.e., age, physical fitness level, health status, working status). Another limitation of this study is the selected time for the implementation of the program. The implementation of workplace exercise programs outside the working hours secures the successful participation of the employees without interfering with their working responsibilities and obligations. Thus, for employees with an increased workload and increased working obligations, the design and implementation of the workplace program outside the working hours maybe the most ideal approach. However, the selection of the most appropriate and effective time (before or after the working hours) for the implementation of the program as well as the adaptation of the exercise interventions (i.e., training load characteristics, exercise activities) to the professional work characteristics are of crucial importance. For example, participation in a training program (approximately 50–60 min) immediately before the working hours, as some employees carried it out, may affect their work efficiency (at least at the beginning of the work shift). Furthermore, the incorporation of a circuit training program into a workplace exercise program (immediately before or after the work shift) maybe more demanding, although our participants were trained and the training load characteristics were adjusted so that they did not affect their working efficiency. However, in the present study, we did not examine the impact of the exercise program on the employees’ efficiency. Finally, another limitation of this study is the test used for the evaluation of dynamic balance and general functional capacity “time up and go” in both the exercise and control groups before and after the 4-month period. Although the “time up and go test” is widely used as a simple and low-cost test for dynamic balance and general functional capacity assessment, it has some limitations. The “time up and go test” is mainly used for middle-aged and elderly individuals as well as in individuals with chronic diseases, while it is not so specialized for young and mainly trained individuals. The use of an appropriate test for dynamic balance and general functional capacity assessment, according to the subjects’ characteristics found in the present study (age and training status), could give a clearer picture regarding the efficacy of the program on dynamic balance improvement.

## 5. Conclusions

In conclusion, the results of the present study have important practical applications, indicating that a 4-month combined yoga, Pilates, and circuit strength training program outside the work shift (before or after the working hours) may be an enjoyable intervention to improve specific health, functional capacity (flexibility, balance), and physical fitness indices (strength). However, the integration of a specific aerobic exercise modality into this program should be seen as important in order to induce significant improvements in cardiorespiratory fitness.

## Figures and Tables

**Table 1:** Age, demographic, and anthropometric characteristics of the sample (mean ± standard deviation).

Characteristics	Training Group (*n* = 25)	Control Group (*n* = 25)
Gender	8 males; 17 females	9 males; 16 females
*Age (years old)*	38.6 ± 8.4	38.0 ± 8.5
*Body mass (kg)*	68.5 ± 16.4	69.3 ± 18.4
*Body height (m)*	1.69 ± 0.1	1.70 ± 0.1
BMI (kg/m^2^) *	23.6 ± 3.4	23.9 ± 3.6

* BMI: body mass index = body mass/body height^2^.

**Table 2:** Gradual increase in the training load during flexibility, balance, and strength workouts.

	Months			
	1	2	3	4
**Flexibility (Yoga program)**				
Routines/sequences	Surya Namaskar sequence A (10 poses) Surya Namaskar sequence B (19 poses)	Surya Namaskar sequence A (10 poses) Surya Namaskar sequence B (19 poses)	Surya Namaskar sequence B (19 poses) Moon Salutation Chandra Namaskar (17 poses)	Surya Namaskar sequence B (19 poses) Moon Salutation Chandra Namaskar (17 poses)
Number of sets per routine	2	2–3	3–4	4
Duration per pose (s)	15	15–20	20–25	25–30
Rest per set/routine (min)	2	2	1.5	1
**Balance (Pilates program)**				
Number of sets per sequence	2	2–3	3–4	4
Repetitions per exercise/ Duration per exercise (s)	12/ 15	12–15/ 15–20	15–20/ 20–25	20/ 25–30
Rest per set/sequence (min)	2	2	1.5	1
**Strength (Circuit training)**				
Number of rounds per session	3	3	4	4
Rest per round (min)	2	2	1.5	1
Duration per exercise (s)	20	20–25	20–25	25–30

**Table 3:** An indicative training session (the 10th training session) of the 4-month intervention program.

	Training Contents	Training Variables
**Warm-up**	Running drills Static and dynamic stretching	15 min
**Main part**		30 min
Circuit strength training	Low Row with Trx (lats and back muscles) Isometric Squat holding medicine ball (extensor muscles of the lower extremity, shoulders) Russian Twists with medicine ball (abdominal muscles) Front Squat with dumbbells (extensor muscles of lower extremity) French press with dumbbell (triceps, arms) Trx Mountain Climbers (abdominal muscles, shoulders, arms) Push-ups (chest, arms, shoulders) Superman plank (lats, back muscles)	Volume: 8 exercises, 3 cycles Duration: 10 min/cycle, 20 sec/exercise Break: 2 min/cycle
Or Yoga and Pilates training	Yoga (Sun salutation yoga sequences) Surya Namaskar sequence A (10 poses) Surya Namaskar sequence B (19 poses) Pilates: Static balance routine (exercises while standing with body weight and using a swiss ball).	Volume: 2 cycles sequence Duration/Reps: 15 s/pose (yoga)/12 reps/exercise (Pilates) Break: 2 min/cycle sequence
**Cool down**	Static stretching for the whole body	5 min

**Table 4:** Employees’ health indices per group (TG and CG) and measurement (pre and post) (means ± SDs).

Variables	Group	Pre	Post	% Mean Change
Body mass (kg)	TG	68.5 ± 16.4	65.9 ±16.4 *^,#^	−4.4 ^†^
	CG	69.3 ± 18.4	69.8 ± 18.9	+0.7
Body fat (%)	TG	16.3 ± 7.9	14.4 ± 7.2 *^,#^	−6.5 ^†^
	CG	15.6 ± 8.7	15.9 ± 8.8	+1.9
Hip circumference (cm)	TG	96.9 ± 9.5	93.6 ± 8.6 *^,#^	−3.6 ^†^
	CG	96.7 ± 9.1	96.6 ± 9.5	−0.1
Waist circumference (cm)	TG	82.8 ± 11.9	80. 6 ± 11.4 *^,#^	−2.8 ^†^
	CG	81.8 ± 13.3	81.9 ± 14.0	+0.1

Where * *p* < 0.01 = a statistically significant difference between before and after the intervention program in the TG, ^#^ *p* < 0.01 = a statistically significant difference between the TG and CG in the post-training measurement, and ^†^ *p* < 0.01 = a statistically significant difference between the TG and CG in percent change. TG: training group, CG: control group.

**Table 5:** Musculoskeletal pain indices per group (TG and CG) and measurement (pre and post) (means ± SDs).

Variables	Group	Pre	Post	% Mean Change
Duration of pain (number of days)	TG	45.8 ± 24.2	20 ± 10 *^,#^	−56.3 ^†^
	CG	44.1 ± 23.9	46.9 ± 33.6	+5.9
Intensity of pain (0–10 scale)	TG	6.8 ± 3.4	3.8 ± 1.4 *^,#^	−44.1 ^†^
	CG	6.6 ± 3.3	6.6 ± 3.4	0
Absenteeism from work (number of days)	TG	5.4 ± 4.1	2.7 ± 0.9 *^,#^	−50 ^†^
	CG	5.6 ± 4.7	5.7 ± 5	+1.8
Negative impact of musculoskeletal pains in daily activities (number of days)	TG	9.7 ± 6.5	4.7 ± 1.1 *^,#^	−51.5 ^†^
	CG	9.9 ± 6.6	10 ± 6.5	+1

Where * *p* < 0.001 = a statistically significant difference between before and after the intervention program in the TG, ^#^ *p* < 0.001 = a statistically significant difference between the TG and CG in the post-training measurement, and ^†^ *p* < 0.001 = a statistically significant difference between the TG and CG in percent change. TG: training group, CG: control group.

**Table 6:** Flexibility and balance values per group (TG and CG) and measurement (pre and post) (means ± SDs).

Variables	Groups	Pre	Post	% Mean Change
Sit and reach test (cm)	TG	24.4 ± 7.9	31.9 ± 7.1 *^,#^	+24.7 ^†^
	CG	26.2 ± 7.8	26.5 ± 8.2	+1.1
Back scratch test (cm) Right hand	TG	6.9 ± 10.2	13.5 ± 10.1 *^,#^	+48.9 ^†^
	CG	7.4 ± 10.1	7.6 ± 9.4	+2.6
Left hand	TG	1.4 ± 12.1	7.6 ± 10.9 *^,#^	+82 ^†^
	CG	1.8 ± 13.5	1.9 ± 12.3	+1.2
Static balance (s) Right leg	TG	18.1 ± 5.9	28.6 ± 5.6 *^,#^	+36.3 ^†^
	CG	18 ± 6.2	18.3 ± 6.1	+1.6
Left leg	TG	15.8 ± 6.4	24.1 ± 6.6 *^,#^	+35.1 ^†^
	CG	15.7 ± 6.9	16.3 ± 6.7	+3.3
Dynamic balance TUG test (s)	TG	5.5 ± 0.9	5 ± 0.9 *^,#^	−11.3 ^†^
	CG	5.2 ± 0.8	5.3 ± 0.8	+1.9

Where * *p* < 0.001 = a statistically significant difference between before and after the intervention program in the TG, ^#^ *p* < 0.001 = a statistically significant difference between the TG and CG in the post-training measurement, and ^†^ *p* < 0.001 = a statistically significant difference between the TG and CG in percent change. TG: training group, CG: control group.

## Data Availability

Data are unavailable due to privacy or ethical restrictions.

## Supplementary Materials

The following supporting information can be downloaded at: https://www.mdpi.com/article/10.3390/sports11040084/s1, Table S1: Consensus on Exercise Reporting Template (CERT) checklist; Table S2: Flexibility, balance and strength exercises.
