# Supplementary material for: An Enjoyable Workplace Combined Exercise Program for Health Promotion in Trained Employees: Yoga, Pilates, and Circuit Strength Training
Source: Sports (Basel). 2023 Apr 14;11(4):84. doi: 10.3390/sports11040084 (PMC10145485; doi:10.3390/sports11040084)
Supplement: Supplementary file 1 [file sports-11-00084-s001.zip › sports-2247030-supplementary.pdf]

Supplementary Table S1. Consensus on Exercise Reporting Template (CERT) checklist.

Section/Topic	Item #	Checklist item	Location<br>Primary paper (page, lines, table etc.)
**WHAT: materials**	1	Detailed description of the type of exercise equipment (e.g. weights, exercise equipment such as machines, treadmill, bicycle ergometer etc).	page 4, lines 144-149
**WHO: provider**	2	Detailed description of the qualifications, teaching/supervising expertise, and/or training undertaken by the exercise instructor	page 4, lines 121-122
**HOW: delivery**	3	Describe whether exercises are performed individually or in a group	page 4, lines 135-136
	4	Describe whether exercises are supervised or unsupervised and how they are delivered	page 4, lines 121-122
	5	Detailed description of how adherence to exercise is measured and reported	page 4, lines 123-124
	6	Detailed description of motivation strategies	page 4, lines 124-126
	7a	Detailed description of the decision rule(s) for determining exercise progression	page 4, lines 152-154; Table 2
	7b	Detailed description of how the exercise program was progressed	page 4, lines 152-154; Table 2
	8	Detailed description of each exercise to enable replication (e.g. photographs, illustrations, video etc)	supplementary table 2
	9	Detailed description of any home program component (e.g. other exercises, stretching etc)	N/A
	10	Describe whether there are any non-exercise components (e.g. education, cognitive behavioural therapy, massage etc)	N/A
	11	Describe the type and number of adverse events that occurred during exercise	page 3, line 118
**WHERE: location**	12	Describe the setting in which the exercises are performed	page 4, lines 135-136
**WHEN, HOW MUCH: dosage**	13	Detailed description of the exercise intervention including, but not limited to, number of exercise repetitions/sets/sessions, session duration, intervention/program duration etc	page 4-6, lines 128-172; table 2; table 3; supplementary table 2
**TAILORING: what, how**	14a	Describe whether the exercises are generic (one size fits all) or tailored whether tailored to the individual	page 6, lines 163-172
	14b	Detailed description of how exercises are tailored to the individual	page 6, lines 163-172
	15	Describe the decision rule for determining the starting level at which people commence an exercise program (such as beginner, intermediate, advanced etc).	page 6, lines 163-172
**HOW WELL: planned, actual**	16a	Describe how adherence or fidelity to the exercise intervention is assessed/measured	page 4; lines 123-124
	16b	Describe the extent to which the intervention was delivered as planned	N/A

Supplementary Table S2. Flexibility, balance and strength exercises.

**Flexibility (using yoga exercise workout)**	
**Sequence - Routine**	**URL**
Surya Namaskar sequence A (10 poses)	Sun Salutation A (Surya Namaskar A) - Raj Yoga Rishikesh
Surya Namaskar sequence B (19 poses)	Sun Salutation B || Surya Namaskar B – ILONABARNHART
Moon Salutation Chandra Namaskar (17 poses)	Traditional Chandra Namaskar (Moon Salutation) — The authentic way - Complete Wellbeing
**Balance - Stability training (using Pilates exercise workout)**	
**Sequence - Routine - Exercise**	**URL**
Standing balance exercises with body weight	1. https://youtu.be/-oAKtBt8tyE<br>2. https://youtu.be/4yqTMoe7A48
Stability exercises with swiss ball	1. ACE - ProSource™: September 2015 - Programming Spotlight: Stability and Mobility Training With the Stabi... | Stability ball, Stability ball exercises, Ball exercises (pinterest.com)<br>2. How To Do Stability Ball Lunge (gethealthyu.com)<br>3. Premium Photo | Good looking girl balancing while lying on a fit ball with arms and legs extended inside her house. (freepik.com)<br>4. stability exercises with swiss ball - Bing images<br>5. https://co.pinterest.com/pin/684687949570736147/
**Strength training (using circuit exercise program)**	
**Exercise**	**URL**
Low Row with Trx	Low Row with Trx - Bing images
Isometric Squat Holding Medicine Ball	Isometric Squat Holding Medicine Ball - Bing images
Russian Twists with medicine ball	russian twists exercise with medicine ball - Bing images
Front Squat with dumbbells	Front Squat with dumbbells - Bing images
French press with dumbbell	french press workout dumbbell - Bing images
Trx Mountain Climbers	TRX Mountain Climber | Trx ejercicios, Ejercicios, Trx (pinterest.com)
Push-ups	push-ups exercise - Bing images
Superman plank	Superman plank - Bing images
